# Enhanced Arabic disaster data classification using domain adaptation

**DOI:** 10.1371/journal.pone.0301255

**Published:** 2024-04-04

**Authors:** Abdullah M. Moussa, Sherif Abdou, Khaled M. Elsayed, Mohsen Rashwan, Amna Asif, Shaheen Khatoon, Majed A. Alshamari

**Affiliations:** 1 The Engineering Company for the Development of Digital Systems, Giza, Egypt; 2 Faculty of Computers and Artificial Intelligence, Cairo University, Giza, Egypt; 3 Faculty of Engineering, Cairo University, Giza, Egypt; 4 School of Computing and Communications, Lancaster University Leipzig, Leipzig, Germany; 5 School of Architecture, Computing & Engineering, University of East London, London, United Kingdom; 6 College of Computer Sciences and Information Technology, King Faisal University, AlAhsa, Saudi Arabia; American University of Beirut, LEBANON

## Abstract

Natural disasters, like pandemics and earthquakes, are some of the main causes of distress and casualties. Governmental crisis management processes are crucial when dealing with these types of problems. Social media platforms are among the main sources of information regarding current events and public opinion. So, they have been used extensively to aid disaster detection and prevention efforts. Therefore, there is always a need for better automatic systems that can detect and classify disaster data of social media. In this work, we propose enhanced Arabic disaster data classification models. The suggested models utilize domain adaptation to provide state-of-the-art accuracy. We used a standard dataset of Arabic disaster data collected from Twitter for testing the proposed models. Experimental results show that the provided models significantly outperform the previous state-of-the-art results.

## Introduction

All over time, the world faces many types of crises. Natural crises, like floods, earthquakes, and tsunamis, can have huge negative impacts and causalities. The recent earthquake that hit eastern Turkey and northern Syria is a typical example of what a natural disaster can do. In just minutes, the earthquake yields tens of thousands of causalities and thousands of destroyed buildings. Other examples of disasters include the COVID-19 pandemic and the major wildfires that hit Canada recently.

Due to the large scale of natural crises impacts, significant efforts are dedicated to reducing the massive impacts of disasters. Several governmental and humanitarian authorities are established to provide rescue efforts for refugees and other disasters’ victims. In times of disasters, every minute is precious when dealing with new information about the problem. Decision makers need to have all the important disaster-related information in the minimum time possible. Several sources of data can be used to retrieve such information. For example, social media is one of these main sources. A typical behavior of social media users, in times of a disaster, is to keep updating their followers with important details about new incidents. For instance, within a large-scale earthquake, one user may provide his followers with the location of a specific destroyed building that needs emergent response to help in rescuing the collapsed building residents. However, such vital information can be easily neglected due to the huge amounts of data generated on social media platforms. So, there is always a need for automatic tools that can efficiently gather, analyze, and classify important disaster-relevant social media data, allowing for better time utilization.

Recent advances in artificial intelligence in general and machine learning in particular were utilized to enhance how to develop disaster-handling automatic tools [1]. The new developments recently proposed are also helpful when dealing with man-made crises [2, 3]. Recent improvements in machine learning solutions, especially deep learning-based ones, provide a significant boost when developing such tools, thanks to their robust classification capabilities. Given a large amount of social media texts, deep learning-based neural networks can classify the texts into several predefined classes. For example, a neural network can automatically classify a post about a group of people who are trapped in a house during a flood as an important disaster-relevant event, and classify a tweet like "Messi shots are more dangerous to the fans than Corona." as a disaster-irrelevant tweet.

While the English datasets of natural disasters are relatively frequent, there is a shortage of Arabic language-based disaster datasets. In [4], the authors propose Kawarith, a multi-dialect Arabic-based natural disasters corpus that is collected from Twitter. The dataset was about 22 disasters that occurred between 2018 and 2020 and included seven types of crises. One of them is a global crisis which is COVID-19, while the other six are disasters like floods and bombings that happened in some Arabic countries. The dataset consists of two parts, a labeled part and an unlabeled one. Each tweet in the labeled part is associated with one of seven labels such as warnings, damage to infrastructure, and irrelevant tweets. The dataset is intended to be used by the disaster research community as a benchmark reference. It can be used to test and compare proposed disaster-related data classification models. It is worth mentioning that Twitter is of special importance when dealing with instant responses from social media users because the platform is designed to encourage users to update their followers with brief important information. It also encourages the usage of hashtags to make it easier for users to access tweets that match their interests. These features make Twitter a very good source when searching for disaster-related crucial information. However, we should mention that in many scenarios, Twitter may be of limited usage since just a small percentage of tweets are geo-located which may not be very helpful in some large-scale disasters. So, using Twitter data would be more helpful if it is associated with other efforts and data collection.

In this work, we used the Kawarith dataset to provide enhanced Arabic disaster data classification models. We utilized the domain adaptation technique to provide the current state-of-the-art accuracies. The provided results illustrated that the suggested models significantly outperform the previous results by a large margin.

The organization of the rest of this work is as the following: The Related work section provides the related work of the problem. The Proposed work section illustrates the -presented work and models. In the Experimental results section, the results of the conducted experiments are given. And finally, conclusions—are shown in the Conclusions and discussions section.

## Related work

Many techniques utilized for aiding the management of disasters rely on methods that are based on machine learning. Techniques of machine learning include Naïve Bayes methods, support vector machine, logistic regression, and neural networks (NN). The main type of neural networks used recently, thanks to its remarkable performance, is deep learning-based NN. Deep learning techniques include several types of neural networks that differ mainly in architecture and have a common feature of including many hidden layers in their networks. Some examples of these varieties are recurrent NN, long short term memory NN (LSTM), convolution-based NN, and transformers architecture [5–7].

Natural Language Processing (NLP) is one of the main branches that makes use of the recent deep learning advances. Many new NLP models have been proposed using deep learning–based techniques, and several of them are based on transformers architecture. A good example of this type of models is BERT [8]. The following subsections present the main characteristics of BERT and some of the other similar BERT-based models that target the Arabic language.

### BERT model

**Fig 1:**
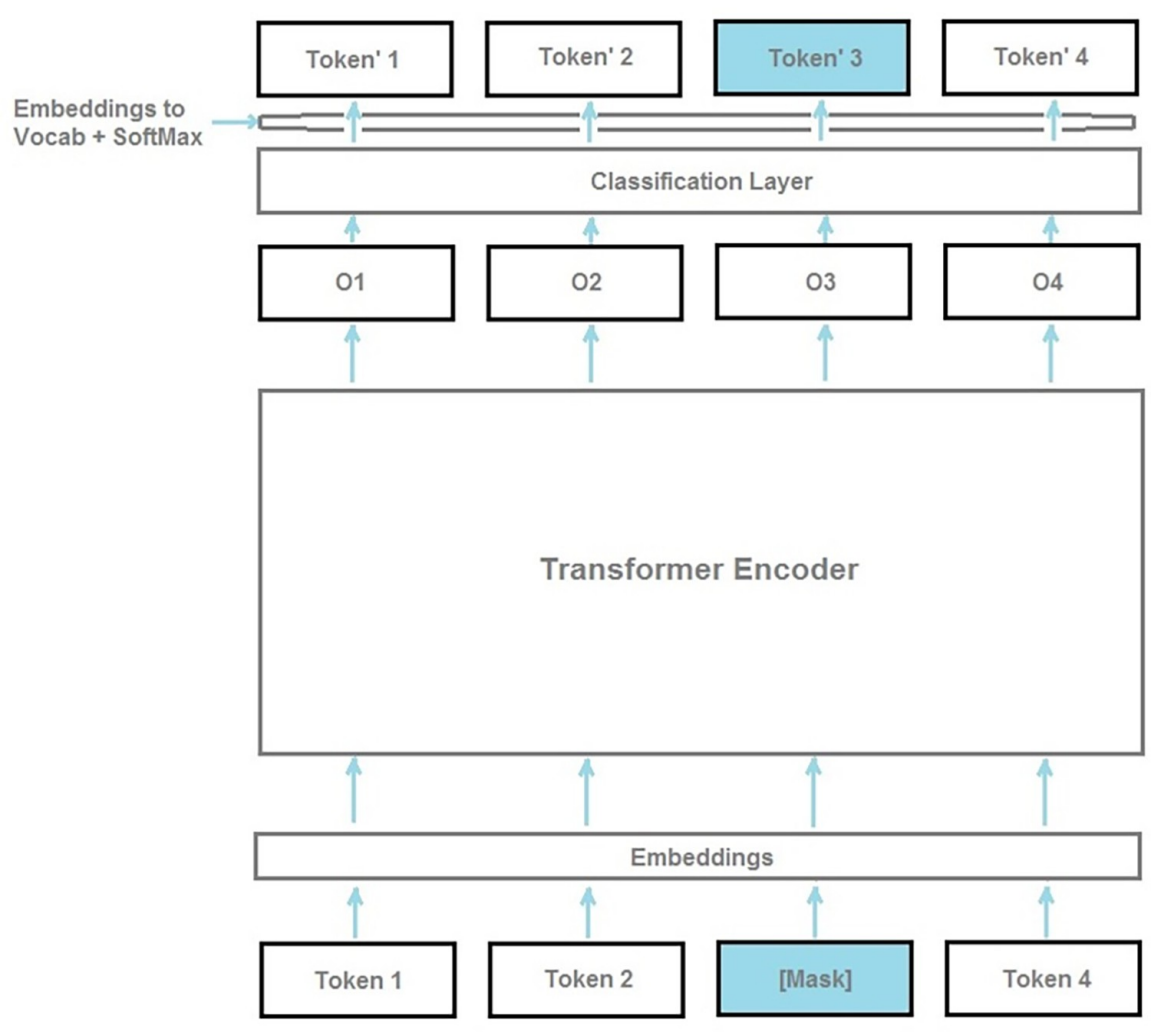
The BERT model structure [9].

Bidirectional Encoder Representations from Transformers (BERT) [8] is a neural network architecture designed for pretraining in natural language processing (Fig 1). The BERT procedure is based on masked language modeling that corrupts the input by replacing a subset of the input tokens with MASK and then trains a neural network model to reconstruct the original tokens. BERT utilizes unlabeled corpus to train bidirectional representations, by jointly conditioning the left and right context among all the network layers.

By adding output layer, the resulting model can be fine-tuned to achieve state-of-the-art performance on a wide range of NLP tasks. In the proposed work, BERT-based models have been fine-tuned to provide text-based emergency-related label classification models.

In the conducted experiments, we fine-tuned three different pre-trained BERT models. One of them (AraBERT) is used to replicate the results of [10] on the retrieved dataset. The following is a summary of the pre-trained BERT models we used.

### AraBERT

After the success of the English version of BERT, The authors of [10] proposed AraBERT as a pre-trained model dedicated to Arabic. It has state-of-the-art results on several tasks of Arabic NLP. AraBERT has been trained by using a dataset that has been crawled from websites of Arabic news and some other resources of Arabic. The AraBERT model outperforms the multilingual BERT version proposed by Google (mBERT) in many downstream tasks. The AraBERT model specifications are illustrated in Table 1.

**Table 1:** Different large-scale Arabic language models.

	Number of attention heads	Number of hidden layers	Hidden size	Size of vocabulary
**AraBERT**	12	12	768	64,000
**MARBERT**	12	12	768	100,000
**ARBERT**	12	12	768	100,000

### MARBERT

MARBERT [11] is a BERT-based large-scale Arabic language model. The dataset used to train MARBERT consists of a large amount of Arabic tweets. It targets both dialectal and MSA Arabic. It has the best performance when used with dialectal Arabic downstream tasks. In several of such tasks, MARBERT has state-of-the-art results. The main characteristics of MARBERT are shown in Table 1.

### ARBERT

ARBERT was proposed with MARBERT in [11] to target MSA Arabic. ARBERT was trained on a large amount of MSA Arabic texts including Wikipedia Arabic and Gigaword. The general specifications of the model are presented in Table 1.

To train a robust deep learning model, the training process should make use of large datasets. To utilize the new advances proposed by deep learning in disaster detection and rescue efforts, several disaster datasets have been proposed in the English language [12–18]. However, there is a relative shortage of similar datasets for the Arabic language. In [4], the authors proposed Kawarith, an Arabic-based disaster dataset collected from Twitter as a good trial to provide important data resources for Arabic-based disaster-related research efforts. The main characteristics of the dataset are illustrated in the following subsection.

### Kawarith dataset

In [4], the authors introduced Kawarith, a corpus retrieved from Twitter Arabic tweets that are multi-dialect. The corpus was built to represent disaster events, containing around one million Arabic tweets that are gathered at the times of twenty-two different crises that happened between 2018 and 2020. The dataset targets medium to high-risk disasters that are most probably to initiate considerable social media activity. It also contains a range of crisis sorts, like bombing, floods, shootings, pandemics, sandstorms, wildfires, and explosions. Using NLP techniques, the authors identified the types of related information published on Twitter. Also, their work proposed a labeled dataset comprising six labels that can be used as a gold standard for many tasks in disaster handling research. The manually annotated subset contains more than 12k tweets from seven different crises. These crises are Jordan floods, Kuwait floods, Cairo bombing, Hafr Albatin floods, Dragon storms, Covid-19, and Beirut explosion. Each annotated tweet, except tweets within the Covid-19 subset, is associated with one of the following labels:

- Affected persons & seeking help
- Damage of Infrastructure
- Warnings and updates of the crisis
- Emotional support and prayers
- Criticism
- Irrelevant tweets

For the Covid-19 dataset, the label is either Relevant or Irrelevant. Table 2 illustrates several examples of the tweets in Kawarith and their labels.

**Table 2:** Some examples of tweets in Kawarith with their labels.

Label	Tweet
Affected persons & seeking help	بيروت محافظ بيروت؛ يدور علي المرفا ويبكي فقط يبكي قائلا في ناس هون مفقودين هون في ناس بحبن متل ولادي بيروت Beirut, Beirut governor is checking Beirut harbor and crying. He is crying and saying: here, there are missing people. People I love like my children.
Damage of Infrastructure	مصر صحيفه الخليج عاصفه غير مسبوقه تضرب مصر وتتسبب في تجمعات مياه الامطار في العديد من الشوارع والميادين صحيفه الخليج Egypt, The Gulf Newspaper, Exceptional storm hits Egypt and causes collections of rains water in many streets and squares.
Warnings and updates of the crisis	حفرالباطن سحب غزيره كاسره في اتجاه حفرالباطن الان Hafr Albatin, Many clouds are going towards Hafr Albatin now.
Emotional support and prayers	سيول الاردن اللهم احفظ الاردن وشعبها. Jordan floods, May Allah save Jordan and its people.
Criticism	سيول الاردن اعداد الوفيات في تزايد تعتيم غير مبرر هل لان الحادثه لا تخص العاصمه عمان سيول الاردن Jordan floods, The number of deaths is increasing, There is unjustifiable secrecy. Is this because the accident is not related to the capital Amman?
Irrelevant tweets	تسديدات كريستيانو اخطر علي الجماهير من كورونا Cristiano kicks are more dangerous to the fans than Corona.

During a crisis, it is critical to monitor the related information on social media. Several efforts were proposed for this objective [1], for example, Fan et al. [19] proposed results of detecting events of disasters from posts of social media by using machine learning techniques. The authors suggested a machine learning model that uses tweets related to a specific crisis to discover crisis-related events. A BERT model was used to tackle posts classification problem.

In [12] the authors utilized online news to monitor disasters. Machine learning-based techniques have been used in their work to detect useful data using irrelevant data filtration. The classification of text has been conducted by utilizing supervised machine learning techniques that identify news data which are gathered from various stories and articles. The proposed technique can be used to track crisis-related online news to improve crisis response.

In another work, the authors of [17] proposed a crisis management system by using NLP and machine learning models. A scraping method was utilized to scrape news that is relevant to crises from various resources of news, and NLP and machine learning methods were utilized to specify the crises-related data. The identified parts of the data were shared with the crisis management community. For gathering news from various news websites, a spider-scraper method was utilized. Their proposed technique segmented the news dataset by using machine learning into disaster-irrelevant and disaster-relevant news which were shared for aiding crisis management efforts.

While many efforts have been made to support disaster detection and rescue efforts using the English language, and due to the relative shortage of Arabic disaster-related datasets, there is still a need for more efforts to support Arabic-based disaster management applications. In [4], the authors provided Twitter-based Arabic disaster datasets and proposed benchmark classification models that can classify their presented data into a specific predefined number of disaster-related labels. In the following section, we illustrate our proposed models that significantly outperform the models presented in [4] in terms of both accuracy and hamming loss.

## Proposed work

In our work, we used the Kawarith dataset [4]. The labeled part of the dataset consists of seven groups of tweets; each group represents a specific one of the crises and is segmented into a training section and a testing section. Due to the regulations of Twitter, only tweet IDs of a dataset can be released, so we used the IDs of the tweets published by the authors of [4] to retrieve the tweets’ text. Table 3 illustrates the details of retrieved tweets of the labeled part of Kawarith. Similarly, we retrieved the unlabeled part of Kawarith. It consists of about 7.6M words with a considerable amount of repeated tweets. Notice that due to the inaccessibility of some tweets, for example, some tweets may have been deleted or their tweeting accounts have been suspended, the numbers of retrieved tweets are different from the numbers stated in [4].

**Table 3:** Details of the retrieved labeled part of Kawarith.

year	crisis name	country	start date	# training tweets	# testing tweets
2018	Jordan floods	Jordan	25/10/18	1160	282
2018	Kuwait floods-18	Kuwait	04/11/18	2346	595
2019	Cairo bombing	Egypt	04/08/19	451	116
2020	Dragon storms	Egypt	12/03/20	628	159
2020	Beirut explosion	Lebanon	04/08/20	648	148
2019	Hafr Albatin floods-19	Saudi Arabia	25/10/19	869	199
2019	Covid-19	Worldwide	01/12/19	1080	266

To have a robust classification system for different types of emergency-related labels, we need a background model that can represent tweets language. While the majority of tweets in the dataset are written using dialectal Arabic, there is a need for a reliable dialectal Arabic language model. We chose to use MARBERT [11]. We also utilized ARBERT [11] along with retesting AraBERT. In [4], The AraBERT model has been used to provide a benchmark for Kawarith. We reapplied the model in our experiments due to the mentioned difference in the retrieved number of tweets between [4] and our work.

Background BERT-based models should not be utilized directly for text classification. So given a BERT-based model, a labeled training dataset can be used for the fine-tuning of the model. The input to the model is a tweet text associated with a label and the output of the model should be compared to the objective label to minimize the difference in order to fine-tune the model weights. The result of the process is an emergency-related label classification model. Once we have the fine-tuned model, we can query it by using a tweet text and the model would suggest an emergency-related label for our input.

We conducted two groups of experiments. In the first group, the base models, MARBERT, AraBERT, and ARBERT, were converted to emergency-related label classification models using the labeled tweets datasets. i.e., each one of MARBERT, AraBERT, and ARBERT is separately transformed into a label classification model using the labeled training data of each dataset listed in Table 3. Based on the fact that the tweets in COVID-19 dataset have only one of two labels, Relevant or Irrelevant, the models generated using this dataset are binary classifiers. And the models generated using the other datasets are 6-label classifiers that can distinguish between the mentioned six labels listed in Kawarith.

**Fig 2:**
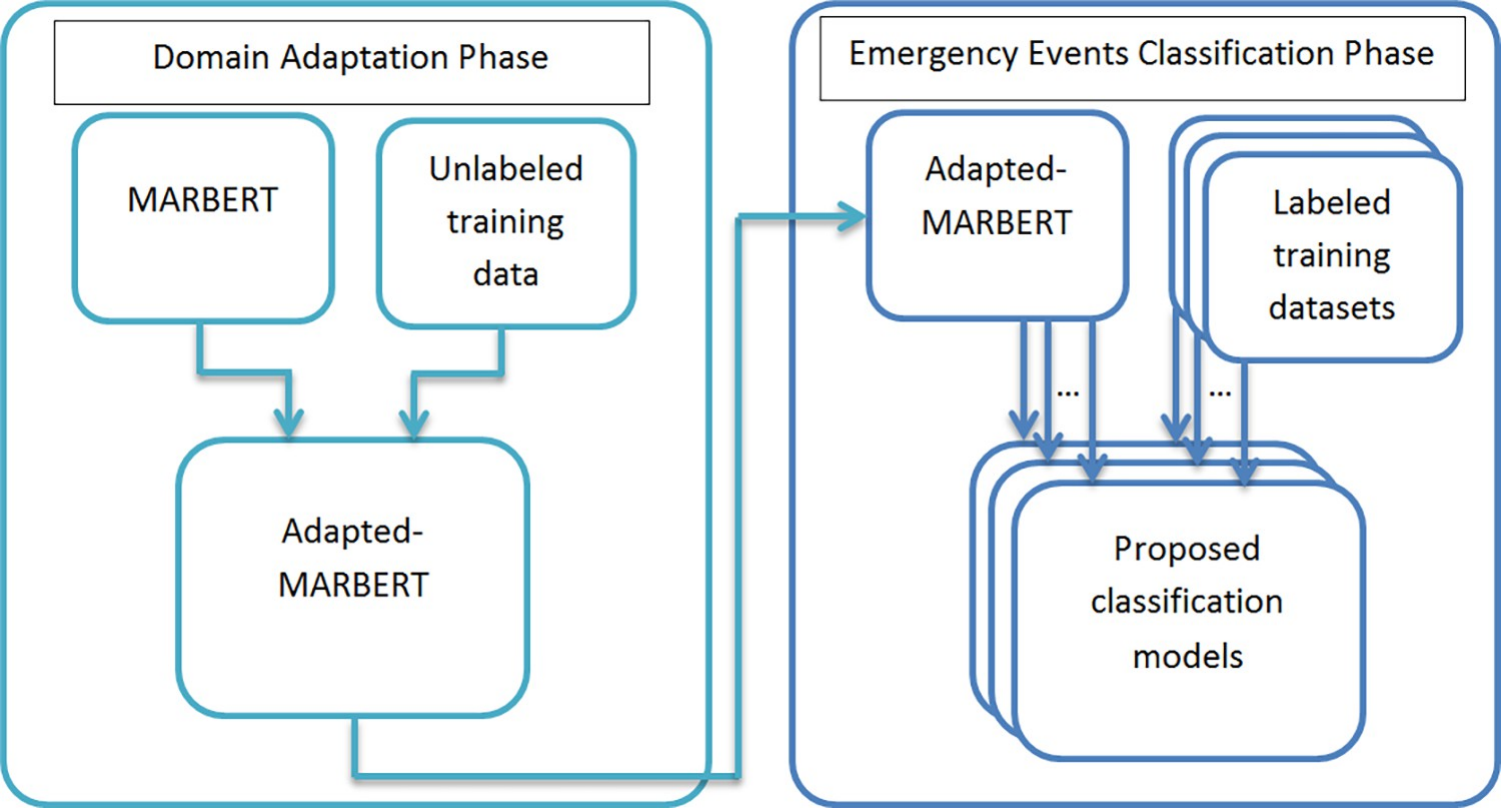
The main methodology of the proposed work.

In the second group of experiments, we applied two phases. First, we utilized the unlabeled part of Kawarith to produce a fine-tuned version of MARBERT using a self-supervised mechanism. In phase two, we converted the resulting adapted-MARBERT to emergency-related label classification models using the criteria mentioned in the first group of experiments. Fig 2 illustrates the main methodology of the second group of experiments.

## Experimental results

The following is an illustration of the obtained results after applying the experiments. Table 4 shows the results of the first group of experiments. The table illustrates the details of the experiments conducted using labeled datasets shown in Table 3. We changed the batch size of the experiment with the values 8, 32, and 64 for each conducted experiment. The illustrated results are in terms of accuracy. The used accuracy metric is the total number of correct hits divided by the total number of all predictions. For each dataset, the previous state-of-the-art score [4], which is retested on the retrieved datasets, and the newly achieved score are highlighted.

**Table 4:** Experiments details for the first group of experiments.

dataset	base model	batch size	best epoch result (accuracy)
Beirut explosion	bert-base-arabert (previous state-of-the-art [4])	8	**0.716**
Beirut explosion	bert-base-arabert	32	0.696
Beirut explosion	bert-base-arabert	64	0.676
Beirut explosion	ARBERT	8	0.223
Beirut explosion	ARBERT	32	0.75
Beirut explosion	ARBERT	64	0.743
Beirut explosion	MARBERT	8	0.73
Beirut explosion	MARBERT	32	**0.784**
Beirut explosion	MARBERT	64	0.777
Jordan floods	bert-base-arabert	8	0.787
Jordan floods	bert-base-arabert	32	0.791
Jordan floods	bert-base-arabert (previous state-of-the-art [4])	64	**0.809**
Jordan floods	ARBERT	8	0.784
Jordan floods	ARBERT	32	0.809
Jordan floods	ARBERT	64	0.83
Jordan floods	MARBERT	8	0.77
Jordan floods	MARBERT	32	**0.833**
Jordan floods	MARBERT	64	0.823
Kuwait floods	bert-base-arabert	8	0.297
Kuwait floods	bert-base-arabert (previous state-of-the-art [4])	32	**0.765**
Kuwait floods	bert-base-arabert	64	0.758
Kuwait floods	ARBERT	8	0.733
Kuwait floods	ARBERT	32	0.761
Kuwait floods	ARBERT	64	0.775
Kuwait floods	MARBERT	8	0.187
Kuwait floods	MARBERT	32	0.787
Kuwait floods	MARBERT	64	**0.795**
Hafr Albatin floods	bert-base-arabert	8	0.704
Hafr Albatin floods	bert-base-arabert (previous state-of-the-art [4])	32	**0.779**
Hafr Albatin floods	bert-base-arabert	64	0.447
Hafr Albatin floods	ARBERT	8	0.759
Hafr Albatin floods	ARBERT	32	0.794
Hafr Albatin floods	ARBERT	64	0.804
Hafr Albatin floods	MARBERT	8	0.794
Hafr Albatin floods	MARBERT	32	0.804
Hafr Albatin floods	MARBERT	64	**0.829**
Dragon storms	bert-base-arabert	8	0.088
Dragon storms	bert-base-arabert	32	0.711
Dragon storms	bert-base-arabert (previous state-of-the-art [4])	64	**0.723**
Dragon storms	ARBERT	8	0.296
Dragon storms	ARBERT	32	0.761
Dragon storms	ARBERT	64	0.742
Dragon storms	MARBERT	8	0.604
Dragon storms	MARBERT	32	0.755
Dragon storms	MARBERT	64	**0.78**
Cairo bombing	bert-base-arabert	8	0.692
Cairo bombing	bert-base-arabert (previous state-of-the-art [4])	32	**0.761**
Cairo bombing	bert-base-arabert	64	0.384
Cairo bombing	ARBERT	8	0.201
Cairo bombing	ARBERT	32	0.723
Cairo bombing	ARBERT	64	0.723
Cairo bombing	MARBERT	8	0.723
Cairo bombing	MARBERT	32	0.767
Cairo bombing	MARBERT	64	**0.767**

As we can see in Table 4, there are significant absolute gains achieved when using MARBERT-based classification models. The best-proposed models have absolute increases in accuracy of 6.8%, 2.4%, 3.0%, 5.0%, 5.7%, and 0.6% over state-of-the-art results for Beirut explosion, Kuwait floods, Jordan floods, Dragon storms, Hafr Albatin floods and Cairo bombing datasets respectively. This demonstrates that the usage of a dialectal base model has a remarkable effect on results.

Table 5 shows the achieved results of COVID-19 dataset experiments. We tested different values of batch size, dropout, max length of used text, and learning rate as illustrated in the table. As mentioned before, while each tweet in the datasets illustrated in Table 3 has one of the different six labels, the tweets in the Covid-19 dataset have either Relevant or Irrelevant labels. This makes the accuracies of both the state-of-the-art and the suggested model relatively higher than the accuracy of models in Table 4 because the generated models are binary classifiers. This also would explain that the absolute gain obtained by the best proposed model was 0.7% as there is no large room for significant enhancement.

**Table 5:** Experiments details of Covid-19 dataset.

base model	dropout	max length	batch size	learning rate	best epoch result (accuracy)
bert-base-arabert	0.2	60	64	5.00E-05	0.94
bert-base-arabert	0.2	60	32	5.00E-05	0.936
bert-base-arabert (previous state-of-the-art [4])	0.2	60	8	5.00E-05	**0.944**
MARBERT	0.2	110	64	5.00E-05	0.936
MARBERT	0.2	110	32	5.00E-05	0.936
MARBERT	0.2	110	16	5.00E-05	0.936
MARBERT	0.2	110	8	5.00E-05	0.921
MARBERT	0.2	180	55	5.00E-05	0.932
MARBERT	0.2	180	50	5.00E-05	0.947
MARBERT	0.2	180	32	5.00E-05	0.94
MARBERT	0.2	256	32	5.00E-05	0.944
MARBERT	0.2	256	16	5.00E-05	0.929
MARBERT	0.2	220	32	5.00E-05	0.932
MARBERT	0.2	160	64	5.00E-05	0.944
MARBERT	0.2	160	32	5.00E-05	0.94
MARBERT	0.2	200	50	5.00E-05	0.944
MARBERT	0.2	200	32	5.00E-05	0.925
MARBERT	0.2	60	50	5.00E-05	0.944
ARBERT	0.2	180	50	5.00E-05	0.947
ARBERT	0.2	180	32	5.00E-05	0.94
ARBERT	0.2	60	50	5.00E-05	**0.951**
ARBERT	0.2	60	64	5.00E-05	**0.951**
ARBERT	0.2	60	80	5.00E-05	0.936
ARBERT	0.2	60	32	5.00E-05	0.947
ARBERT	0.3	60	64	5.00E-05	0.944
ARBERT	0.1	60	64	5.00E-05	0.947
ARBERT	0.15	60	64	5.00E-05	0.951
ARBERT	0.2	60	64	5.00E-04	0.91
ARBERT	0.2	60	64	5.00E-06	0.947
ARBERT	0.2	60	64	5.00E-03	0.91
ARBERT	0.2	60	64	5.00E-07	0.91
ARBERT	0.2	80	64	5.00E-05	0.944
ARBERT	0.2	50	64	5.00E-05	**0.951**

### Domain adaptation experiments

In the second group of experiments, we tested the effect of using MARBERT after applying domain adaptation. In each experiment, the unlabeled part of Kawarith was used to adapt the MARBERT model. This process was applied for a different number of epochs spanning from one to five. The resulting models were then converted to classification models using the labeled datasets in Table 3. Table 6 presents the details of the experiments.

**Table 6:** Details of domain adaptation-based experiments.

Dataset	Model	batch size	best epoch result (accuracy)
Beirut explosion	MARBERT-adapted /epoch_1	32	0.736
Beirut explosion	MARBERT-adapted /epoch_2	32	**0.791**
Beirut explosion	MARBERT-adapted /epoch_3	32	0.75
Beirut explosion	MARBERT-adapted /epoch_4	32	0.709
Beirut explosion	MARBERT-adapted /epoch_5	32	0.723
Jordan floods	MARBERT-adapted /epoch_1	32	**0.826**
Jordan floods	MARBERT-adapted /epoch_2	32	0.801
Jordan floods	MARBERT-adapted /epoch_3	32	0.812
Jordan floods	MARBERT-adapted /epoch_4	32	0.812
Jordan floods	MARBERT-adapted /epoch_5	32	0.805
Kuwait floods	MARBERT-adapted /epoch_1	64	0.797
Kuwait floods	MARBERT-adapted /epoch_2	64	0.795
Kuwait floods	MARBERT-adapted /epoch_3	64	0.79
Kuwait floods	MARBERT-adapted /epoch_4	64	**0.802**
Kuwait floods	MARBERT-adapted /epoch_5	64	0.795
Hafr Albatin floods	MARBERT-adapted /epoch_1	64	0.804
Hafr Albatin floods	MARBERT-adapted /epoch_2	64	0.789
Hafr Albatin floods	MARBERT-adapted /epoch_3	64	0.794
Hafr Albatin floods	MARBERT-adapted /epoch_4	64	0.789
Hafr Albatin floods	MARBERT-adapted /epoch_5	64	**0.809**
Dragon storms	MARBERT-adapted /epoch_1	64	0.553
Dragon storms	MARBERT-adapted /epoch_2	64	0.723
Dragon storms	MARBERT-adapted /epoch_3	64	0.723
Dragon storms	MARBERT-adapted /epoch_4	64	0.73
Dragon storms	MARBERT-adapted /epoch_5	64	**0.742**
Cairo bombing	MARBERT-adapted /epoch_1	64	**0.828**
Cairo bombing	MARBERT-adapted /epoch_2	64	0.793
Cairo bombing	MARBERT-adapted /epoch_3	64	0.802
Cairo bombing	MARBERT-adapted /epoch_4	64	0.784
Cairo bombing	MARBERT-adapted /epoch_5	64	0.81

While the unlabeled dataset used in the experiment was of small size, Table 6 shows that introducing the domain adaptation-based solution enhanced the accuracy of three models out of the six models. Specifically, the accuracy of the domain adaptation-based model for the Beirut explosion dataset was enhanced by 0.7% of absolute gain. We also achieved absolute accuracy gains of 0.7% and 6.1% for the Kuwait floods and Cairo bombing models respectively. Table 7 summarizes a comparison of the previous state-of-the-art accuracies against the achieved accuracies of the suggested models.

**Table 7:** Summary of the achieved results based on accuracy.

Dataset	previous state-of-the-art [4] (based on accuracy)	Proposed score	Absolute gain
Beirut explosion	0.716	0.791	7.5%
Jordan floods	0.809	0.833	2.4%
Kuwait floods	0.765	0.802	3.7%
Hafr Albatin floods	0.779	0.829	5%
Dragon storms	0.723	0.78	5.7%
Cairo bombing	0.761	0.828	6.7%
Covid-19	0.944	0.951	0.7%

As we can see in Table 7, the calculated accuracies of the best proposed models show significant gains over state-of-the-art models. We also calculated the hamming loss for the best proposed models against state-of-the-art models. Table 8 illustrates the computed scores.

**Table 8:** Summary of the proposed results based on hamming loss.

Dataset	previous state-of-the-art [4] (based on Hamming loss)	Proposed score	Absolute gain
Beirut explosion	0.047	0.035	1.2%
Jordan floods	0.032	0.028	0.4%
Kuwait floods	0.039	0.033	0.6%
Hafr Albatin floods	0.037	0.028	0.9%
Dragon storms	0.046	0.037	0.9%
Cairo bombing	0.039	0.029	1.0%
Covid-19	0.056	0.049	0.7%

As shown in Table 8, the proposed models provide better hamming losses (the less the better) over state-of-the-art models for all datasets. Obtaining gains in terms of accuracy and hamming loss demonstrate the enhanced performance of the proposed models.

## Conclusions and discussions

Some of the main sources of disturbance and casualties are natural hazards. Governments all over the world are in continuous need of more robust detection and handling processes of such crises. Classification of emergency-related text on social media streams can provide vital information to governmental efforts when facing a natural disaster. In this work, an emergency-related label classifier was proposed based on an Arabic Twitter dataset focused on disasters that happened in several Arabic countries. Experimental results showed that the proposed models are significantly better in terms of accuracy and hamming loss than state-of-the-art results.

Despite the significant results proposed in this work, there are some limitations to be considered. For example, the proposed models relied only on text, while social media streams are using images and videos beside text to represent events in general and disasters in particular. Also, although the dataset used in the work is recent and focused on disasters, however, the size of the dataset is not large. So, there is a need for larger datasets for robust testing of the proposed models’ reliability. Our future work includes training a unified classification model using all the labeled data. We also consider integrating the proposed models with other disaster image and video-based classification models for full utilization of different types of useful related information.

## Supporting information

S1 File The code used in the implementation. (ZIP)

S2 File The data used in the work. (ZIP)
